# Smoothed particle hydrodynamic modelling of the cerebrospinal fluid for brain biomechanics: Accuracy and stability

**DOI:** 10.1002/cnm.3440

**Published:** 2021-02-09

**Authors:** Harry Duckworth, David J. Sharp, Mazdak Ghajari

**Affiliations:** ^1^ Dyson School of Design Engineering Imperial College London London UK; ^2^ The Computational, Cognitive and Clinical Neuroimaging Laboratory Imperial College London London UK; ^3^ Care Research and Technology Centre Dementia Research Institute London UK

**Keywords:** brain biomechanics, cerebrospinal fluid, finite element modelling, smoothed particle hydrodynamics

## Abstract

The Cerebrospinal Fluid (CSF) can undergo shear deformations under head motions. Finite Element (FE) models, which are commonly used to simulate biomechanics of the brain, including traumatic brain injury, employ solid elements to represent the CSF. However, the limited number of elements paired with shear deformations in CSF can decrease the accuracy of their predictions. Large deformation problems can be accurately modelled using the mesh‐free Smoothed Particle Hydrodynamics (SPH) method, but there is limited previous work on using this method for modelling the CSF. Here we explored the stability and accuracy of key modelling parameters of an SPH model of the CSF when predicting relative brain/skull displacements in a simulation of an in vivo mild head impact in human. The Moving Least Squares (MLS) SPH formulation and Ogden rubber material model were found to be the most accurate and stable. The strain and strain rate in the brain differed across the SPH and FE models of CSF. The FE mesh anchored the gyri, preventing them from experiencing the level of strains seen in the in vivo brain experiments and predicted by the SPH model. Additionally, SPH showed higher levels of strains in the sulci compared to the FE model. However, tensile instability was found to be a key challenge of the SPH method, which needs to be addressed in future. Our study provides a detailed investigation of the use of SPH and shows its potential for improving the accuracy of computational models of brain biomechanics.

## Graphical abstract

Here we show the key benefits, and challenges, when modelling brain biomechanics using the meshfree method Smoothed Particle Hydrodynamics (SPH) to represent the cerebrospinal fluid in finite element models. A parametric study of the accuracy and stability of different modelling parameters is presented. Additionally, the meshfree method is shown to predict strains and strain rates similar to those seen in‐vivo, unlike the traditional modelling method.

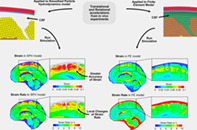

## 1 INTRODUCTION

Finite Element (FE) brain models are commonly used to assess brain biomechanics and the link between clinical pathology and brain deformation under head motion, for example, in traumatic brain injury (TBI).^1^, ^2^, ^3^, ^4^ Head motion can lead to relative displacement between the brain and skull, as seen in in vivo experiments in primate and human.^5^, ^6^, ^7^ Subdural haemorrhaging, often caused by the rupture of bridging veins under high strains, is strongly associated with large relative displacements between the brain and the skull.^8^, ^9^, ^10^

The pia‐arachnoid complex (PAC) exchanges the loading between the skull and brain and consists of the meninges (dura matter, arachnoid, and pia matter), arachnoid granulations, veins, arteries, sinuses, and the water‐like CSF. Most FE models simplify this complex down to a single part with material properties and contact definitions which represent the combination of features present.^11^ They usually refer to this complex as CSF.

In the vast majority of head and brain FE models, the CSF is modelled using solid elements.^11^ Most head models use a tied or tie‐break condition for the CSF, with other models accounting for the relative motion by defining slip conditions.^3^ However, the large shear deformations expected to occur in CSF can have a negative impact on the accuracy of the field variables calculated in some of the CSF solid elements.^3^, ^5^, ^7^, ^12^, ^13^, ^14^, ^15^ To overcome this issue, the Arbitrary Lagrangian–Eulerian (ALE) formulation has also been used to model the CSF.^16^ However, the accuracy of ALE meshes depends on large mesh depths, which can be a problem in modelling the small distance between the skull and brain and brings additional complications to models which contain detailed anatomy such as gyri and sulci.

The meshfree method, Smoothed Particle Hydrodynamics (SPH), holds promise for accurate modelling of the CSF due to its capability to accurately simulate problems that involve large shear deformations. However, there is limited previous work on using this method for modelling the CSF. Wittek et al^17^ compared SPH and FE representation of the CSF in a simplified 2D model of the brain and found that changing the boundary condition method led to differences in the brain/skull relative displacement. A later study showed that a simplified spherical model of the brain with an SPH model of the CSF was able to predict brain/skull relative displacement and CSF pressure to a good level of agreement with the results of impact experiments on a surrogate brain model.^18^ SPH has also been used to model the CSF in a 3‐dimensional model of the human head.^19^ This model, however, showed unrealistic gaps in the SPH in the coup and contrecoup regions under impact loading and lacked comparisons with experimental data. The effects of key SPH modelling parameters on the response of the CSF layer has not been studied before and it is therefore not known how these parameters affect modelling accuracy or stability.

Here, the accuracy and stability of the SPH method in modelling the CSF in an FE model is studied. The SPH formulation, inter‐particle distance and smoothing length are studied to determine their effects on model predictions when compared with the in vivo data from a mild head impact experiment in human (Feng et al^7^). CSF material model and property choice can significantly influence the stability and accuracy of the simulation.^3^, ^14^, ^15^, ^20^ Hence, here the predictions of a large number of CSF material models incorporated in both SPH and FE representations of the CSF are determined. Predictions of brain/skull relative displacement and strain and strain rate distribution across the brain are compared to better understand the stability and accuracy of the SPH versus FE models of CSF.

## 2 METHODS

### 2.1 Smoothed particle hydrodynamics

**FIGURE 1:**
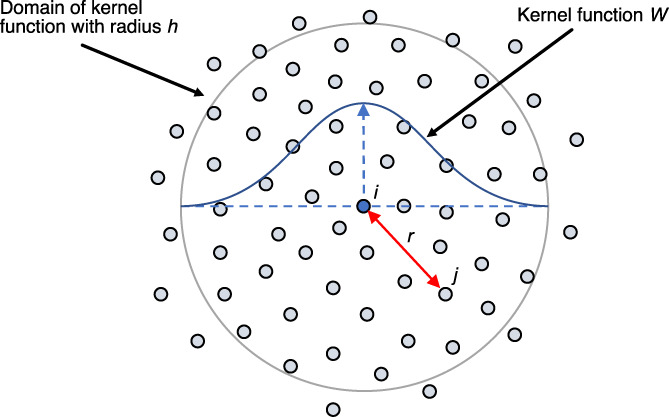
Domain of particle i with kernel function which shows the influence of neighbouring particles. Particle j is a distance r from particle i

SPH is a meshfree computational method capable of accurately modelling material flow over large deformations.^21^, ^22^, ^23^ This study uses the SPH implemented in LS‐DYNA hydro‐code.^24^ The SPH method is implemented through discretisation of a solid or fluid continuum into a finite number of moving elements, more commonly called particles (Figure 1).

For particles to influence each other, a kernel function is defined. The kernel function allows for the physical quantity of a particle to be calculated through summation of this quantity in nearby particles within a radius *h* , known as the smoothing length, weighted with a distance‐based function, *W* . This takes the form of a convolution of *W* in an arbitrary field *A* :

(1) $$A\left(r\right)=\int A\left(r^{\prime }\right)W\left(\left|r-r^{\prime }\right|,h\right)\mathrm{dV}\left(r\prime \right).$$

The approximation of Equation (1) can be made using a Riemann summation over *j* which includes all particles:

(2) $$A\left(r\right)=\sum_{j}V_{j}A_{j}W\left(\left|r-r_{j}\right|,h\right),$$

where *A*_*j*_ is the value of field quantity for particle *j* , *V*_*j*_ is the volume of particle *j* , and ***r*** is the position of particle *j* . For example, if finding density, Equation (2) takes the form of:

(3) $$\rho_{i}=\rho \left(r_{i}\right)=\sum_{j}m_{j}W_{\mathrm{ij}},$$

where the mass of particle *j* , *m*_*j*_ , is calculated as *m*_*j*_ = *ρ*_*j*_ *V*_*j*_ , with *ρ*_*j*_ representing the density, and *W*_*ij*_ = *W*(| ***r***_***i***_ − ***r***_***j***_|, *h*). For further information on formulation and implementation of the SPH method refer to^21^ and Hallquist's^24^ LS‐DYNA theory manual.

### 2.2 The brain model

**FIGURE 2:**
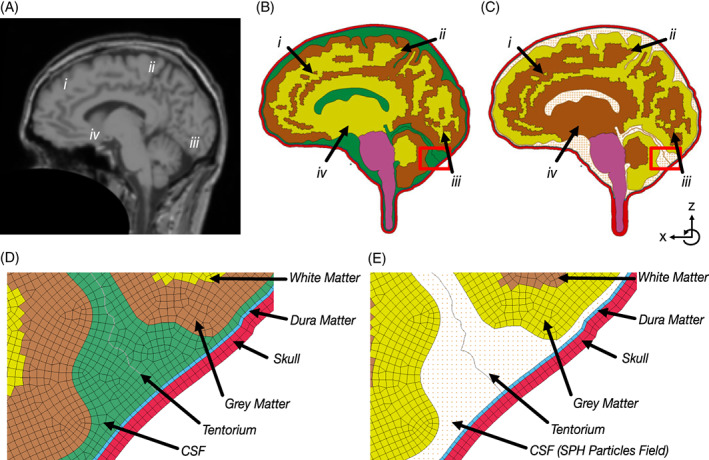
(A) T1 MRI scan of parasagittal plane of subject with approximate data collection locations labelled i–iv, (B) and (C) FE and SPH model with approximate data collection locations labelled i–iv (axes shown in bottom right corner, y axis normal to plane, rotation counter clockwise) (D) and (E) Detail of sulcal and gyral region of FE model and SPH model respectively

A 2D parasagittal slice of the brain, which includes skull, dura, tentorium, grey matter, white matter, brain stem and CSF, was created from a previously developed 3D model of the head and brain^2^ and comparison to MRI scans of the same subject (Figure 2(A)). Two 2D models were created. The first model, henceforth called the FE model, was created using only Lagrangian solid elements (Figure 2(B)). The second model, henceforth called the SPH model, was otherwise identical to the FE model except the solid elements of CSF were replaced with a particle field of SPH elements (Figure 2(C)).

The particle field in the SPH model was made of two distinct parts; the boundary and the grid. The boundary particles were created at the centre of each of the FE boundary elements. To prevent voids forming under tensile forces and to mimic the hydrodynamic boundary layer, these boundary particles were tied to the FE elements using the *TIED_NODES_TO_SURFACE keyword in LS‐DYNA. A uniform grid of particles was chosen to fill the empty space between the brain and the skull, instead of random particle distribution, to reduce noise SPH field variables data.^25^ Seven models were created with inter‐particle distances between 0.2 mm and 2 mm. Any particles which were less than half the interparticle distance from the wall were removed to prevent errors due to abrupt changes in interparticle distance.

Contact was defined using keyword *AUTOMATIC_NODES_TO_SURFACE between the remaining SPH nodes and the boundary walls. All nodes were constrained in the y direction due to negligible displacements reported in the experiment.^7^ The tentorium was created from Belytschko‐Tsay shell elements with three integration points, and the dura was created using solid elements attached to the skull. The skull was modelled as a rigid body and made slave to a solid element placed near the foramen magnum, where accelerations were applied.

#### 2.2.1 Material properties

The material properties of the brain, brainstem, and dura were taken from the previous study which the head and brain models in this study were based on^2^, ^4^ (Appendix A). The material models used for the CSF in the comparison study were elastic, viscoelastic, Ogden rubber, and viscous fluid with an equation of state (Table 1). These were taken from previous studies to represent the several ways authors have represented the CSF in their models.

**TABLE 1:** Material properties from literature used for CSF

Name	Author	Model type	Material properties
Elastic 1	Chafi, Dirisala^25^	Fluid elastic	*ρ* = 1040 kg/m^3^ *ν* = 0.5 *K* = 0.219 GPa *μ* = 0.2
Elastic 2	Chafi, Dirisala^25^	Fluid elastic	*ρ* = 1040 kg/m^3^ *ν* = 0.5 *K* = 0.00219 GPa *μ* = 0.05
Viscoelastic 1	Takhounts, Ridella^26^	Kelvin‐Maxwell Viscoelastic	*ρ* = 1050 kg/m^3^ *K* = 4.96*E* − 03 GPa *G*_0_ = 0.02*E* − 3 GPa *G*_∞_ = 0.02*E* − 3 GPa *β* = 0.01 s^−1^
Viscoelastic 2	Yoganandan, Li^27^	Linear Viscoelastic	*ρ* = 1040 kg/m^3^ *K* = 21.9*E* − 03 GPa *G*_0_ = 0.5*E* − 3 GPa *G*_∞_ = 0.528*E* − 3 GPa *β* = 5 s^−1^
Ogden rubber	Ghajari, Hellyer^2^	Ogden Hyperelasic	*ρ* = 1040 kg/m^3^ *ν* = 0.4998 *μ*_1_ = 20 kPa *α*_1_ = 2
EOS 1	Panzer, Myers^28^	Mie‐Gruneisen equation of State	*ρ* = 1000 kg/m^3^ *C* = 1484 m/s *S*_1_ = 1.979 Γ_0_ = 0.11 *P*_cav_ = − 2.1*E* − 3 GPa* *μ* = 0.8*E* − 9 GPa. ms
EOS 2	Zhou, Li^16^	Mie‐Gruneisen equation of State	*ρ* = 1000 kg/m^3^ *C* = 1482.9 m/s *S*_1_ = 2.1057 *S*_2_ = − 0.1744 *S*_3_ = 0.010085 Γ_0_ = 1.2 *P*_cav_ = − 22*E* − 3 GPa *μ* = 0.001*E* − 9 GPa. m*s*

#### 2.2.2 Loading conditions

An in vivo mild frontal impact on the human head was simulated from experiments conducted by Feng et al.^7^ The experiments consisted of three subjects undergoing mild frontal head impacts inside of an MR scanner, with images taken every 5.6 ms after the head drop was triggered. During the impact experiments, the displacement of the skull and brain in a parasagittal plane were recorded using a tagged MR imaging technique. The motion of the skull was determined through tracking of reference points (Figure 2(A), i–iv). The relative brain/skull motion of four points in the brain were then found by transforming each image to a skull‐fixed coordinate system and calculating the change in the position of brain points relative to the local coordinate system of the skull.

The computational model was loaded with linear and rotational accelerations. The time history of the accelerations were calculated using a five‐point moving average^29^ (Appendix B) as they were not supplied in the original paper. The accelerations were applied to elements in the model near the foramen magnum which represented the centre of gravity. To verify that the calculated accelerations produce the same rigid body displacements as those measured in the experiment, the linear and rotational displacements of the reference point were recorded during the simulations and compared with experimental results.

### 2.3 Parametric study

**FIGURE 3:**
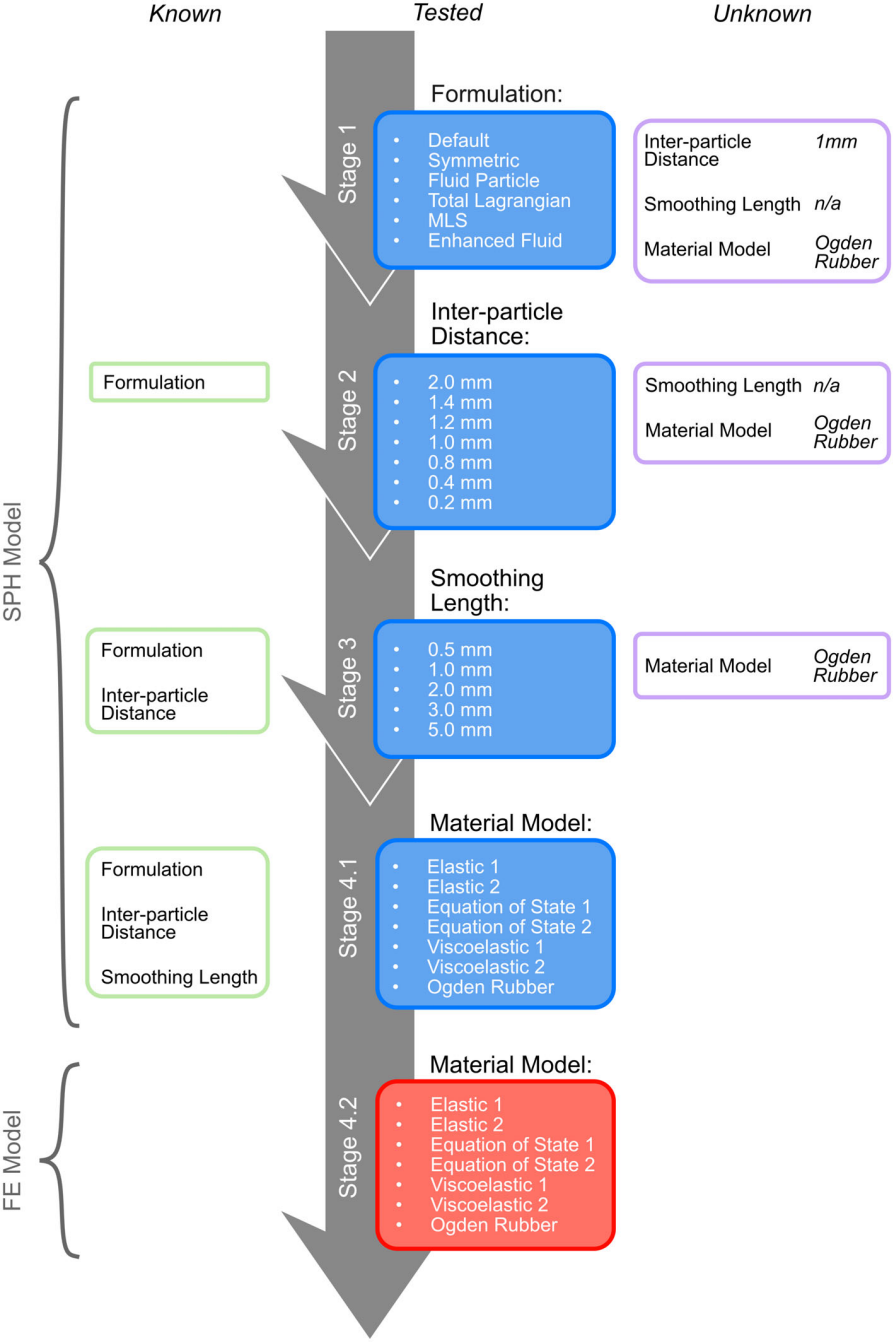
Study design and progression. Default options (shown in the ‘unknown’ column) are chosen for unknown parameters. Central boxes show all options being tests in each stage. Once tested, the parameter which had the highest CORA score and/or was the most stable was set for the next stage of tests. The default smoothing length is allocated by the computer during initialisation

To determine the effects of the SPH properties, four parametric studies were performed: formulation study (stage 1), inter‐particle distance study (stage 2), smoothing length study (stage 3), and material model study (stage 4) (Figure 3). In stage 4, the effects of the material models and properties were studied with both SPH and FE models of the CSF. The order which the studies were carried out may affect the chosen value, so an assumed hierarchy of importance was used based on dependencies, that is, formulation > inter‐particle distance > smoothing length. This was created from insight gained from SPH stability in previous computational tests.

The beta version of LS‐DYNA (R11 at the time of this study) was used for the simulations to ensure some SPH features worked correctly, such as the SPH MLS formulation (FORM = 12). Simulations were run on a high‐performance computer with 20 cores and 36 GB of RAM. A massively parallel processor (MPP) version of LS‐DYNA was used for all simulations.

For each model, brain/skull relative displacement curves measured at four points on the brain were extracted and compared to the experimental data. Tableau Desktop v2019.2.0^30^ was used for data visualisation, and the statistical software package R v3.5.3^31^ was used to analyse results. MATLAB 2018b^32^ was used to process data and generate figures.

### 2.4 Correlation analysis

The correlation analysis (CORA) score implemented in CORA v4.0.4 was used for quantitative comparisons between predicted and experimental time histories of brain displacement (brain displacement locations shown in Figure 2(B, C), i–iv).^33^ CORA is a curve evaluation method, which combines two sub‐methods: a corridor weighting and a cross correlation method to assess similarity between the curves. The corridor weighting calculates the fit of a curve by assigning a value respective to how close a data point is relative to the reference curve, given that it is inside an automatically calculated corridor. The cross‐correlation method uses three categories for phase shift, size and shape of the curve. Combining these two methods together allows for a comprehensive comparison which accounts for key features of the curve. Here we used the default settings of CORA.

A score between 0 and 1 is calculated to represent the model's biofidelity and is categorised as follows: Excellent 0.86–1.0; Good 0.65–0.86; Fair 0.44–0.65; Marginal 0.26–0.44; and Unacceptable 0.0–0.26. These scores are based on the biofidelity ratings found in Technical Report ISO/TR 9790^34^ and have been used in previous studies to rate accuracy of FE models of the brain.^35^, ^36^, ^37^

For each simulation the displacements of the frontal lobe, parietal lobe, occipital lobe, and temporal lobe (specified in Figure 2(B, C), i–iv) were compared to the experimental data using CORA with default values. The final median CORA score was calculated from the four location‐based CORA scores for each simulation to for inter‐model comparison.

## 3 RESULTS

### 3.1 Skull motion and relative brain/skull displacement

**FIGURE 4:**
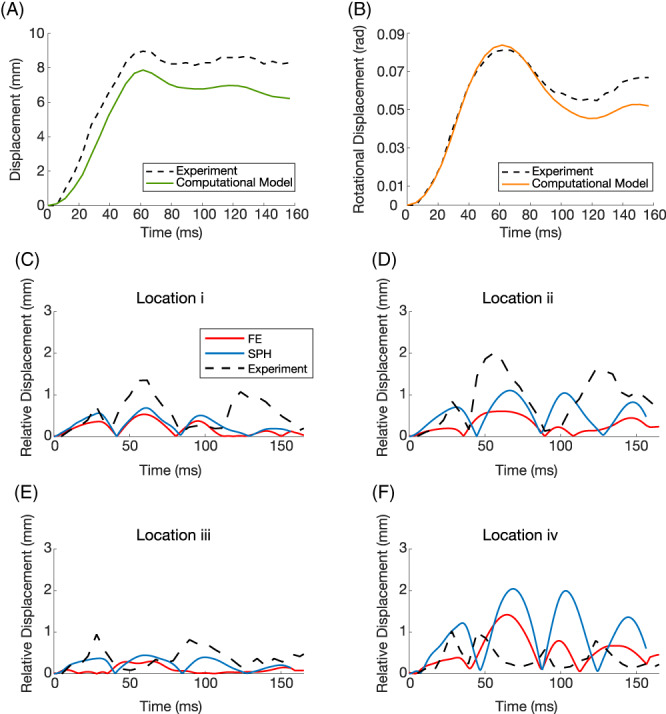
Translational displacement (A) and rotational displacements (B) from experimental data (referred to as S2 in source study), as well as the FE/SPH computation model displacements (dashed line and coloured line respectively), (C–F) relative displacement of brain locations i‐iv for the stage 4 SPH and FE Ogden rubber material model, The average of the CORA score across locations i, ii, iii and iv was 0.40 (marginal) for the SPH model and 0.39 (marginal) for the FEA model

The peak translational and rotational accelerations reported by Feng et al.^7^ were in the range of 14.3–16.3 ms^−2^ and 124–143 rads^−2^ respectively which are higher than those calculated here (7.8 ms^−2^ and 91.1 rads^−2^), which may be related to using different methods for deriving the peak accelerations from the displacement time histories. With our method, however, we found good agreement between the predicted and experimental displacement time histories (Figure 4(A, B)). The CORA scores for rotational and translational displacements are 0.943 (excellent) and 0.819 (good), which confirm the accuracy of the loading conditions.

Figure 4(C–F) shows the predictions of the SPH and FE models using the Ogden rubber material model overlaid on experimental results from Feng et al.^7^ Both models predict the time of the peaks very well in the first 100 ms of the impact for locations i and ii. The models predict smaller, but the SPH model better predicts the displacement in location ii. In locations iii and iv, there is more discrepancy between the predictions and test results. This may be related to the presence of the tentorium and the skull base in close proximity of these locations. The CORA score for location i was 0.449 (fair) for SPH and 0.423 (marginal) for FEA, for ii was 0.343 (marginal) for SPH and 0.471 (fair) FEA, for iii was 0.288 (marginal) for SPH and 0.235 (unacceptable) for FEA and for iv was 0.190 (unacceptable) for SPH and 0.359 (marginal) for FEA. The scores confirm fair/marginal fidelity for locations i and ii and marginal/unacceptable fidelity for locations iii and iv.

### 3.2 The effects of SPH model parameters

#### 3.2.1 SPH formulation

**FIGURE 5:**
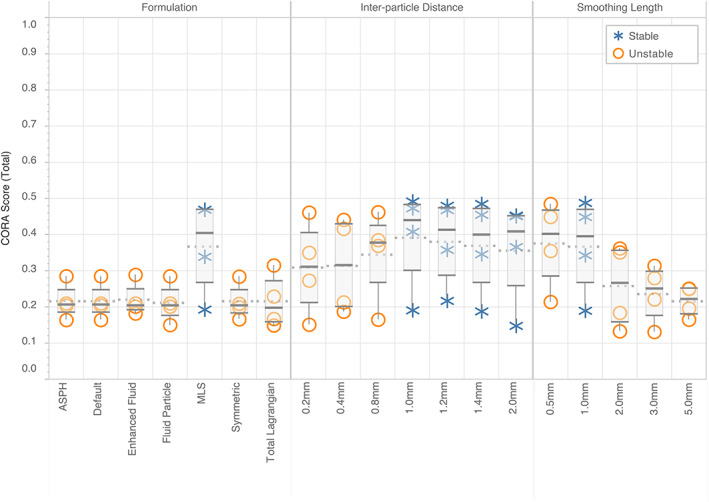
Total CORA scores for each of the locations in the brain for the formulation study, the inter‐particle distance study, and the smoothing length study. Models which were stable are marked with a cross, a circle denotes instability. Tukey box plots (each created from 4 data points) show median, upper and lower hinges, and maximum and minimum data points. The mean for each model is shown with a dotted line

The MLS formulation was stable with a fair CORA score of 0.404 (marginal), with all other formulations showing signs of instability (Figure 5). This was expected as the MLS formulation was created specifically to address the issue of tensile instability.^38^ All other Formulations produced near‐identical results, giving median CORA scores of approximately 0.204 (unacceptable). The MLS formulation had the largest range of CORA scores for locations in the brain, with a maximum spread of 0.276, which is approximately two and a half times greater than the range of values of other formulations.

#### 3.2.2 Inter‐particle distance

The inter‐particle distance study produced a more consistent set of results when compared to the formulation study (Figure 5). Particle densities above 1 mm inclusive were stable and grouped together with median values of approx. 0.420 (marginal). As the inter‐particle distance of 1 mm had the largest CORA score it was chosen for the proceeding studies. The particle densities less than 1 mm showed signs of instability and had the lowest median CORA scores. The range of CORA scores for each location was similar across all particle densities.

#### 3.2.3 Smoothing length

The smoothing length study showed the clearest relationship to CORA score. The smallest smoothing lengths had the largest median CORA score (0.5 mm and 1.0 mm had a similar marginal score of 0.402 and 0.396 each). As smoothing length increased over 1.0 mm, the median CORA score dropped noticeably, with scores of 0.267 for 2 mm (marginal), 0.251 for 3 mm (unacceptable), and 0.223 for 5 mm (unacceptable). The only stable model was that with a smoothing length of 1 mm making it the only choice for the material model study.

#### 3.2.4 Instability

**FIGURE 6:**
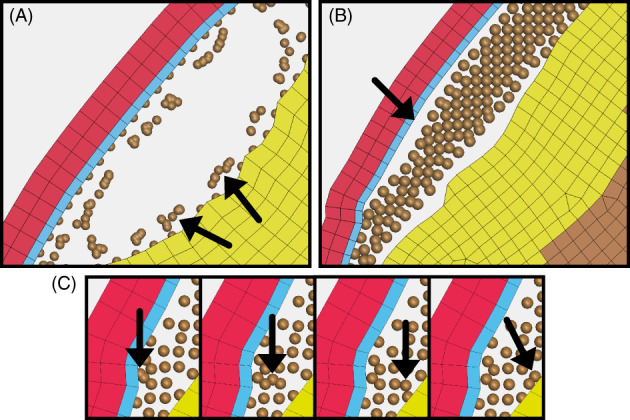
(A) Tensile instability in the SPH. A large void was formed by tensile forces which separate the SPH. Arrows show clumping of particles around boundary, (B) Boundary Instability in the SPH. Separation of the tied SPH elements from the solid elements occurs, shown by the arrow, (C) Deactivation of SPH particles. Arrow shows progress of deactivated particle throughout the domain after deactivation when it reached a threshold velocity. Time step increases in each image from left to right

Three types of instability in the SPH simulations were observed; tensile instability, boundary layer instability, and particle deactivation (Figure 6). Tensile instability is a known limitation of the SPH methods. It occurs when particles under tensile forces separate and stop interacting with each other (Figure 6(A)).^21^, ^39^ This was observed in a large number of simulations (Table 2). This type of instability was difficult to quantitively identify as we knew of no numerical criteria which directly correlated to its occurrence. Tensile instability does have visual characteristics which were looked for in each simulation result. The formation of voids under tensile stresses, spinning clumps of small number of particles around the edges of voids, and unnatural movement of particles pairs of particles were looked for and if identified the simulation was classed as unstable.

**TABLE 2:** The type of instability visually identified in each of the first three studies of the SPH model

Study	Variable	Instability		
		Tensile	Boundary	Deactivation
Formulation	MLS			
	Enhanced Fluid	✗		
	Default	✗		
	Adaptive SPH (ASPH) Anisotropic Smoothing Tensor	✗		
	Total Lagrangian	✗		
	Symmetric	✗		
	Fluid Particle	✗		
Inter‐particle distance	1.0 mm			
	1.2 mm			
	1.4 mm			
	2 mm			
	0.8 mm		✗	✗
	0.4 mm		✗	
	0.2 mm		✗	
Smoothing Length	0.5 mm	✗		
	1.0 mm			
	2.0 mm		✗	✗
	3.0 mm		✗	✗
	5.0 mm		✗	✗

The second form of instability occurred at the boundary of SPH to solid elements (Figure 6(B)). The particles tied to the solid elements were seen to separate from the wall. This was not seen in the formulation study but was common with small particle densities and large smoothing lengths (Table 2). As this instability was visually easy to identify, all models were checked by eye first. Any models where no obvious separation occurred at the boundary of SPH particles to FE mesh had their relative displacements plotted. This allowed for a greater level of accuracy in identification of the presence of instability of ambiguous models.

The third type of instability was observed in the 0.8 mm inter‐particle distance study, and the 2.0 mm, 3.0 mm, and 5.0 mm smoothing length studies. In these simulations, large forces on the SPH particles in the tentorium region resulted in particles exceeding the maximum velocity allowed and they were deactivated (Figure 6(C)). This value was chosen to be 5 m/s after investigation of particle velocities in the simulation which were approximately 10 times less. This value was found to be large enough to not interfere with the stable running of the simulation but small enough to deactivate any particles which reach an unrealistic velocity. Any particles which became deactivated were identified through identification of particles which reached a velocity of 5 m/s.

### 3.3 The effects of CSF material model

**FIGURE 7:**
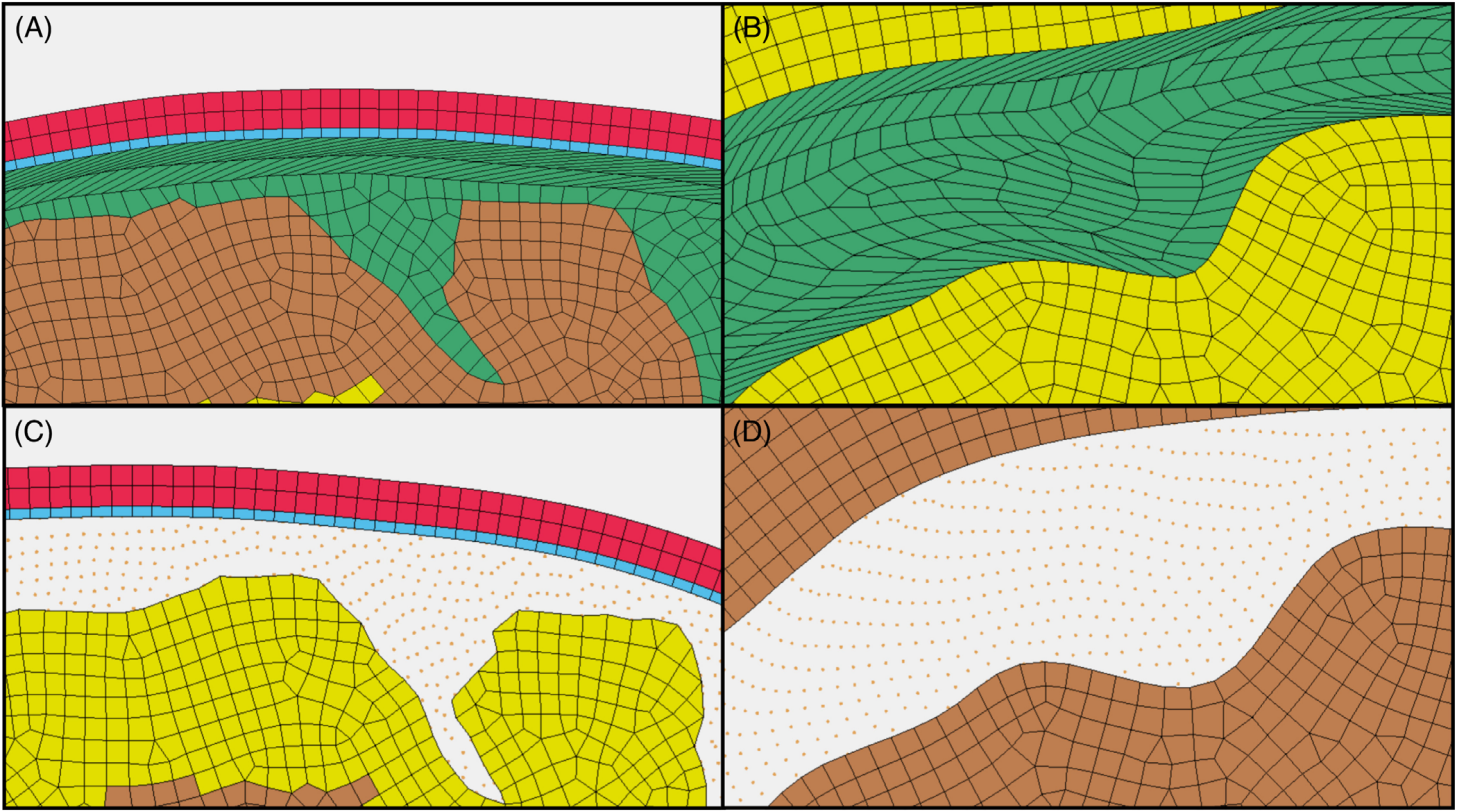
(A) FE model deformation of Viscoelastic 2 material model and (B) deformation in ventricle, (C) SPH deformation of Viscoelastic 2 model and (D) deformation in ventricle

In some material models, the CSF FE elements deformed to a significant extent, leading to excessive distortion of the elements and significant deviation from an ideal element shape (Figure 7(A, B)). The SPH models did not undergo such problems with the high deformations due to the mesh‐free nature of the method (Figure 7(C, D)).

**FIGURE 8:**
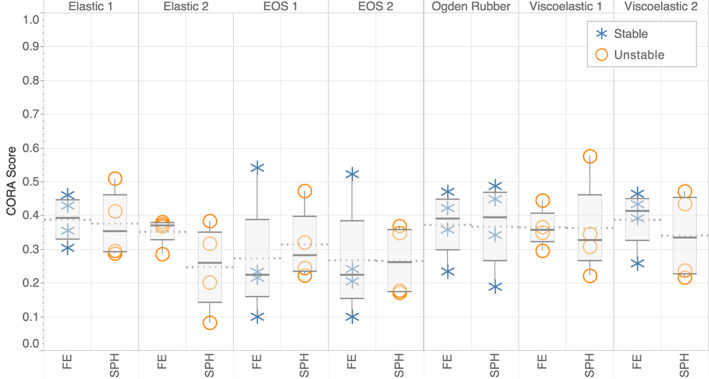
Comparison of total CORA scores for SPH to FE model for each material model. Models which show instability are marked with a circle; a cross denotes stable models. Tukey box plots (each created from 4 data points) show median, upper and lower hinges, and maximum and minimum data points. The mean for each model is shown with a dotted line

The Ogden rubber material model was the only stable model for both the SPH and FE methods. All other SPH material models were unstable, whereas the second Elastic model and the first Viscoelastic model were unstable for the FE model (Figure 8). The Ogden rubber model had similar marginal CORA scores of 0.396 and 0.391 for the SPH and FE models respectively (Figure 4(C–F)). The stable FE models Elastic 1 and Viscoelastic 2 showed marginal CORA scores (around 0.4), but the remaining stable FE models, EOS1 and EOS2, gave the lowest scores, 0.274 and 0.264 respectively (marginal).

#### 3.3.1 Strain and strain rate across the brain

**FIGURE 9:**
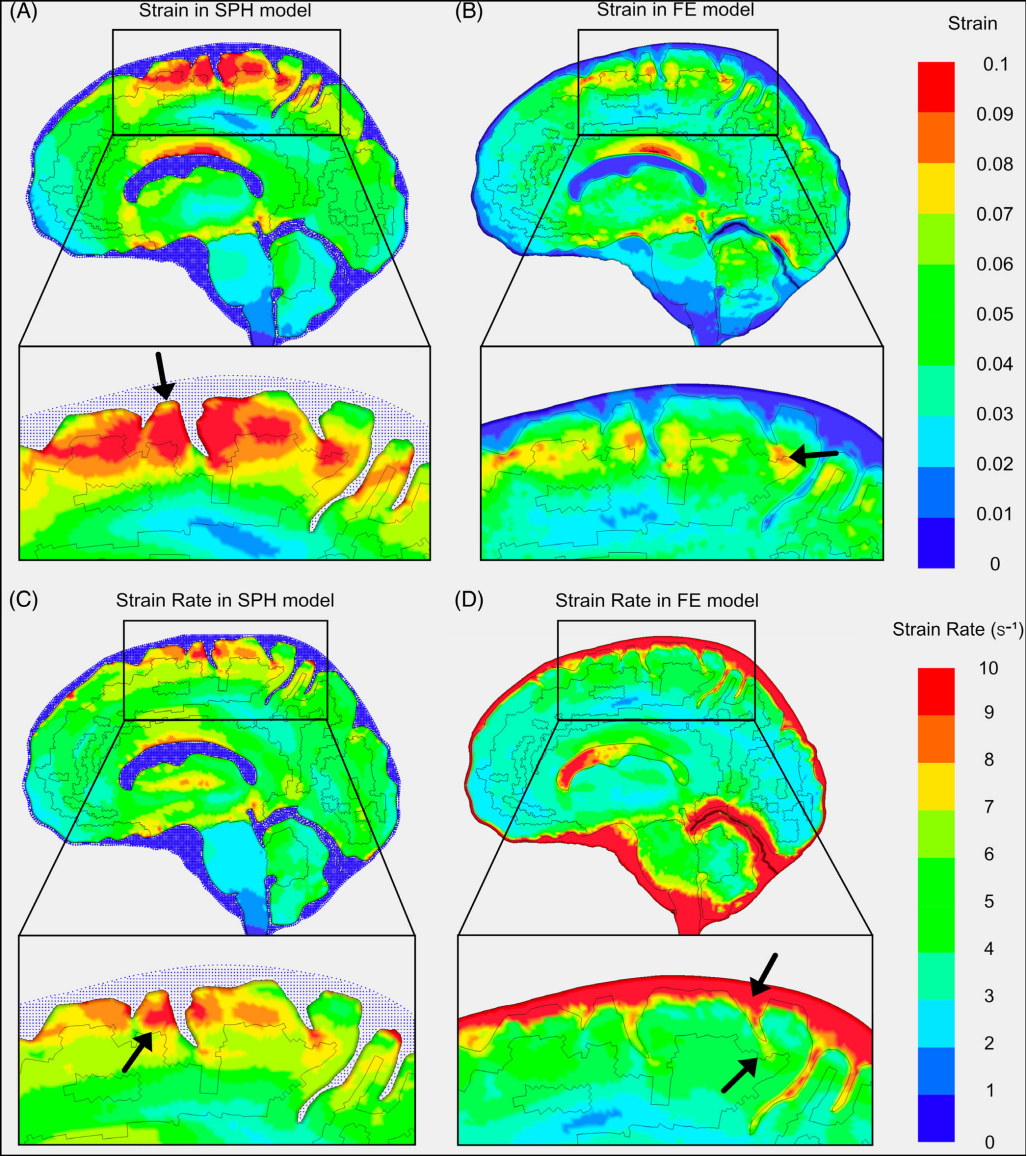
Ogden rubber material models showing: (A) strain in the SPH model (strain in CSF elements not shown), (B) strain in the FE model, (C) strain rate in the SPH model (strain rate in CSF elements not shown), (D) strain rate in the FE model

The maximum value of the first principal Green‐Lagrange strain and strain rate were then compared between the SPH and FE models. The stable Ogden rubber material model for the CSF was compared in order to determine the effects of the modelling technique on biomechanical indicators of tissue injury. The SPH model predicted peak strains of around 0.1 over a sizeable portion of the superior cerebral cortex, including the depths of sulci and some of the gyral regions (Figure 9(A)). The strains reported in these regions are in the same range as those seen the in experiments with peak strains of 0.1 (for whole‐brain strain ellipse plots of experimental data which were used for comparison refer to fig. 9 in Reference 7).

The strains predicted by the FE model were smaller and distributed to a lesser extent, with peak values in the superior cerebral region only occurring in the depths of sulci with maximal values of about 0.08 strain (Figure 9(B)).

There was more noticeable difference between the predicted strain rate distribution using the SPH and FE models (Figure 9). The FE model predicted a nearly uniform distribution of strain rate in the superior region with values around 5 s^−1^ (Figure 9(D)). However, the SPH model predicted strain rates were larger and varying across the superior cerebral region, with a distribution similar to the predicted distribution of strain (Figure 9(C)).

## 4 DISCUSSION

This study evaluates the feasibility of using the mesh‐free SPH method for modelling relative displacements in the CSF during head motions. The relative displacement between brain and skull plays a key role in transferring the external force, particularly its rotational effects, to the brain. By making comparisons to live human data, this study shows that the SPH method has the potential to improve the prediction of relative brain/skull displacement in computational models of brain biomechanics, particularly when detailed anatomy of the brain, such as sulci and gyri, is incorporated in the model.

This study, for the first time, investigates the effects of key SPH parameters on the accuracy of modelling brain/skull relative displacement. The formulation of SPH was found to have the greatest effect on stability and accuracy of the model. The recently developed MLS formulation was found to be the only stable of all available formulations. This formulation was developed to address tensile instability, which was seen when using other formulations. Tensile instability is a key challenge and highly investigated area of the SPH method.^21^, ^39^, ^40^, ^41^, ^42^, ^43^ As the CSF is commonly subjected to tensile forces, particularly in the coup and contrecoup regions, the MLS formulation was shown to provide a stable response within the range of loads studied here.

In this study the SPH particles were coupled with the FE mesh of the brain and skull by using a tied interface. This allowed the SPH method to be used for only the CSF as it undergoes significantly larger shear deformations when compared to the brain. This also avoided formation of voids between CSF and skull, as has been seen in a previous study.^19^ However, this interface choice caused instabilities in some simulations. The coupling method of SPH to FE models is a widely studied area with a number of differing techniques.^44^, ^45^, ^46^, ^47^, ^48^ The simplest SPH to FE interaction method involves constraining the motion of the mesh‐free particles to the nodes or faces of solid elements (the same used in this study). This, however, results in *particle deficiency* at and near the boundary.^21^, ^45^, ^46^ Particles at the boundaries of the SPH domain only have particles inside the boundary interacting with them. This one‐sided interaction results in field variables not being consistent, creating instability in the solution. This was observed when the particle spacing was small and the smoothing length was large, showing that increasing the number of particles inside the domain of a boundary particle can increase the risk of instability. To address this problem, other methods have been created, which use *ghost particles*.^45^, ^47^, ^49^ These particles are pseudo‐elements, which sit outside of the SPH boundary and inside the solid elements. They allow the SPH elements to interact with particles outside the boundary, yet they are only placeholders as they transfer forces to the nodes of the solid elements that they occupy. This more elegant solution allows for a complete domain for the SPH boundary particles, but it is computationally expensive. Attempts were made to model the interface using this method, but due to the large computational requirements and the increase in instability, no models ran successfully. It is recommended to try this interface method after its implementation has been improved.

Ogden rubber was found to be stable for both SPH and FE models and lead to the most accurate predictions out of an extensive range of material models currently being used in literature. Overall, the Ogden rubber material model produced the highest correlations between predictions and experiments with CORA scores around 0.4. This agrees with CORA correlations of other leading models in the field, with average scores found to be marginal, between 0.260 and 0.415 for accuracy of displacements in the brain.^50^

Strain and strain rate distributions were predicted across the brain and in key anatomical regions, such as sulci. The SPH method and its coupling with FE meshes allowed us to incorporate the detailed anatomy of the brain in the model and perform this analysis. The models with SPH and FE representations of the CSF predicted large strains and strain rates in sulcal regions, the location of CTE pathology caused by head impacts.^51^ However, the patterns of strain and strain rate distribution outside the sulcal regions are vastly different, with SPH predicting larger strains in gyral regions. The SPH model predication of strain distribution and magnitude agrees better with strain measurements in the tagged‐MRI human experiment, which have shown that large areas of the superior region of the brain experience strains of 0.1 during the impact.^7^ These results suggest that the FE meshes of CSF act as an anchor for the gyri, reducing the strain and strain rate in these regions. However, the SPH model of CSF allows for more decoupling between CSF and brain, leading to the prediction of larger and more diffuse strains and strain rates across the brain.

One limitation of this study is using a two‐dimensional model, which may influence the prediction of the brain/skull relative displacement. In this study we have compared the results of FEA and hybrid FEA/SPH models of the brain with data from live human experiments.^7^ In the experiments, the head motion was restricted to the sagittal plane and the brain motion in a sagittal slice near the midline was measured. To obtain strain in this slice, plane strain assumption was made. The out of plane motion was shown to have little effect on the validity of results. To replicate these experiments, we also used a plane strain assumption, which allowed us to use a 2D model. Using this approach, which was motivated by previous computational modelling studies,^17^, ^28^, ^52^ allowed us to reduce the simulation time in order to study the effects of a large range of SPH parameters. This study is an important step towards the development of 3D models that can enable predicting brain motion in other locations and under different head impacts. The conclusions of the study are also limited to the mild head loadings studied here. Once the instability of the SPH formulation is addressed, larger head impacts can be studied. This approach however allowed us to compare the predictions with in vivo human data obtained from tagged MRI, providing new insight into the effects of the FE and SPH representations of the CSF on strain and strain rate distribution in cortex.

In summary, this study shows the potential of the SPH mesh‐free method for improving the prediction of brain/skull relative displacement and strain distribution across the brain during head loading. The effects of key SPH parameters, particularly formulation, on the predictions are determined and it is found that tensile instability is a key limitation of the method, which needs to be addressed in future developments and implementations of the SPH method. Nonetheless, the SPH model of CSF provides better predictions of strain distribution than the FE model, during the low acceleration impacts studied here. Accurate modelling of the space between brain and skull is still one of the key challenges of computational biomechanics and using mesh‐free methods for modelling this space is one of the solutions, which we hope will receive a greater level of attention in the coming years with further improvements in their implementation in light of the findings of this study.

## Figures and Tables

**FIGURE B1:**
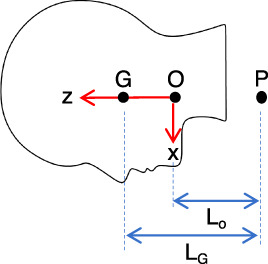
Definition of key points in the neck system; the pivot, P, supports the free rotation of the head with the center of gravity, G, and the origin, O

## Appendix

Table of material properties used for brain model. Dura and brain stem material properties from Reference 2, white and grey matter from Reference 4.

**Table:**

Part	Material Model	Density (kg/mm^3^)	Young's Modulus (GPa)	Poisson's Ratio	
Dura	Elastic	1.130e‐06	0.315	0.45	
White Matter and Grey Matter	Viscoelastic	1.040e‐06			Bulk Modulus = 0.55847 GPa Short Term Shear Modulus = 1.66e‐06 GPa Long Term Shear Modulus = 9.28e‐07 GPa Decay Constant = 0.016 ms^−1^
Brain Stem	Ogden Rubber Hyperelastic	1.040e‐06		0.499982	First Shear Modulus = 1.58e‐08 GPa Second Shear Modulus = 1.58e‐08 GPa First Exponent = 28.1 Second Exponent = −29.5 Shear relaxation moduli and decay constants: *τ*_**1**_ = 1.60000E‐7 ***G***_**1**_ = 0.1 ***τ***_**2**_ = 1.28000E‐5 ***G***_**2**_ = 1 ***τ***_**3**_ = 9.92000E‐6 ***G***_**3**_ = 10 ***τ***_**4**_ = 1.24800E‐4 ***G***_**4**_ = 100 ***τ***_**5**_ = 5.12000E‐4 ***G***_**5**_ = 1000

## Appendix

For calculation of the initial acceleration, the head is modelled as a rigid body with points P, O and G representing the pivot, origin, and centre of gravity respectively (Figure B1). The distances between the pivot and the origin is *L*_*O*_ , and the distance between the pivot and the centre of gravity is *L*_*G*_ .

The translational/rotational acceleration at time *i* is *y*_*i*_^′′^ , with *h* representing the timestep between data points, and *y*_*i* − 2_ , *y*_*i* − 1_ , *y*_*i* + 1_ , and *y*_*i* + 2_ respectively representing the displacements two timesteps before, one timestep before, one timestep after, and two timesteps after the current timestep, *y*_*i*_ .

(B1)
$${\ddot{y}}_{i}=\frac{2y_{i-2}-y_{i-1}-2y_{i}-y_{i+1}+2y_{i+2}}{7h^{2}}.$$

The head is assumed to be near freefall before impact, which is assumed to occur after *t* = 28 ms. This time was found through identification of the point where acceleration begins to decrease significantly.

The sum of the moments about the pivot can be described by (B2), where *W* is the total mass of the head and *I*_*p*_ is its mass moment of inertia about the pivot point. Here it was assumed that the mass and moment of inertia of the fixture is negligible compared to that of the head.

(B2)
$$\sum M_{p}=I_{p}\ddot{\theta }\to WL_{g}=I_{p}\ddot{\theta }.$$

This shows rotational acceleration is constant before the impact. Hence, by integrating (B2), then substituting the constant $\frac{L_{g}W}{I_{p}}=\ddot{\theta }$, the displacement can be described as a function of acceleration (B3).

(B3)
$$\theta =\frac{1}{2}\ddot{\theta }t^{2}\;\left(0<t\leq 28\mathrm{ms}\right).$$

From the experimental data, $\ddot{\theta }$ up to *t* = 28ms was found, which is where minor change in acceleration was seen due to the free‐fall assumption. It was also found that the *x* displacement, for the small rotations which the head undergoes (less than 5°), through assuming that the *x* displacement of the origin was approximately equal to the rotational displacement of the origin from the pivot, *θ* , multiplied by the distance between them *L*_*o*_ (B4).

(B4)
$$x_{0}\approx L_{o}\theta .$$

As *x*_0_ and *θ* are recorded in the experiment, the above Equation (B4) can be used to find *L*_*o*_ = 144mm. Using (B3) and (B4) the initial rotational and translational acceleration can be found. The initial rotational acceleration is found to be *θ*_0 < *t* ≤ 28_ = 91.1rad/s^2^ and the initial translational acceleration in the x direction is found to be *x*_0 < *t* ≤ 28_ = 13.3m/s^2^ . The translational acceleration in the x direction was found to be too large, and therefore was scaled down to *x*_0 < *t* ≤ 28_ = 7.9m/s^2^ to produce the same translational displacement as seen in the experiment the rotational acceleration remained unchanged. This provided the acceleration curves shown in Figure 4(A, B).
